# High fat diet-induced inflammation and oxidative stress are attenuated by N-acetylneuraminic acid in rats

**DOI:** 10.1186/s12929-015-0211-6

**Published:** 2015-10-24

**Authors:** Zhang Yida, Mustapha Umar Imam, Maznah Ismail, Norsharina Ismail, Aini Ideris, Maizaton Atmadini Abdullah

**Affiliations:** Laboratory of Molecular Biomedicine, Institute of Bioscience, Universiti Putra Malaysia, 43400 Serdang, Selangor Malaysia; Cardiology Department, Affiliated Hospital of Chengde Medical University, 067000 Chengde, Hebei China; Department of Nutrition and Dietetics, Faculty of Medicine and Health Sciences, Universiti Putra Malaysia, 43400 Serdang, Selangor Malaysia; Faculty of Veterinary Medicine, Universiti Putra Malaysia, 43400 Serdang, Selangor Malaysia; Department of Pathology, Faculty of Medicine and Health Sciences, Universiti Putra Malaysia, 43400 UPM Serdang, Selangor, Malaysia

**Keywords:** High fat diet, Inflammation, Oxidative stress, N-acetylneuraminic acid, Sialic acid

## Abstract

**Background:**

Serum sialic acid levels are positively correlated with coronary artery disease and inflammation. Although sialic acid is a non-specific marker, it is considered sensitive likely due to its influence in sialylation of glycoprotein structures all over the body.

**Objectives:**

We hypothesized that dietary supplementation with N-acetylneuraminic acid (Neu5Ac), a type of sialic acid, will have profound effects on high fat diet- (HFD-) induced inflammation and oxidative stress in view of the widespread incorporation of sialic acid into glycoprotein structures in the body.

**Methods:**

HFD-fed rats with or without simvastatin or Neu5Ac (50 and 400 mg/kg/day) were followed up for 12 weeks. Lipid profiles, and markers of inflammation (C-reactive protein, interleukin-6, and tumor necrosis factor alpha), insulin resistance (serum insulin and adiponectin, oral glucose tolerance test and homeostatic model of insulin resistance) and oxidative stress (total antioxidant status and thiobarbituric acid reactive species) in the serum and liver were determined, while mRNA levels of hepatic antioxidant and inflammation genes were also quantified. Serum levels of alanine transaminase, aspartate transaminase, alkaline phosphatase, urea, creatinine and uric acid were also assessed.

**Results:**

HFD feeding caused hyperlipidemia and insulin resistance, and worsened liver and kidney functions. HFD feeding also potentiated inflammation and oxidative stress, partly through modulation of hepatic gene expression, while Neu5Ac especially at higher doses and simvastatin attenuated HFD-induced changes, although Neu5Ac showed better outcomes.

**Conclusions:**

Based on the present results, we surmised that Neu5Ac can prevent HFD-induced inflammation and oxidative stress, and may in fact be useful in the prevention of hyperlipidemia-associated inflammation and oxidative stress. However, the translational implications of these findings can only be determined after long-term effects are established. Hence, the use of Neu5Ac on obesity-related diseases requires additional attention.

## Background

Sialic acids (SAs) are small aminosaccharides that consist of a neuraminic acid backbone with one or multiple O- or N-linked side chains, the most abundant of which is N-acetylneuraminic acid (Neu5Ac) in humans. SAs are abundantly present in many human tissues and fluids [1–3], and a positive association between elevated SA levels and coronary artery disease (CAD) has been demonstrated [4–6]. This association may be mediated through CAD risk factors, including changes in serum cholesterol concentrations [7, 8]. Higher levels of SA have also been linked with inflammation [3] and positively correlated with erythrocyte sedimentation rate [9, 10] and the serum concentration of C-reactive protein [8]. Although total serum SA is a sensitive marker of acute phase reactions, it is non-specific because SA is present in many acute phase proteins. On the flip side, there are suggestions that as an acute response marker, SA is simply useful as a substrate for the resialylation of vascular endothelium, in an attempt to reverse atherosclerosis [11]. Despite extensive studies on the implications of endogenous SA levels, not much has been reported on the effects of dietary supplementation on health outcomes.

Studies have indicated that dietary supplementation with N-glycolylneuraminic acid (Neu5Gc) may promote inflammation, hepatocellular cancer and hemolytic-uremic syndrome [12, 13], while supplementation with Neu5Ac has been shown to promote brain development and improve salivation [14, 15]. Based on the widespread presence of Neu5Ac in body fluids and the hypothesis that its elevation as an acute phase marker may be an attempt to enhance the resialylation of vascular endothelium and reduce inflammation, we decided to evaluate its effects on inflammation and oxidative stress in rats. Moreover, there is increasing interest in alternative therapies targeting cardiovascular disease risk factors, including inflammation and oxidative stress. The need for safer and more cost-effective therapies has been the driving force behind this new trend [16].

## Methods

### Materials

Standard rat pellet was purchased from Specialty feeds (Glen Forrest, WA, USA), while cholesterol was purchased from Amresco (Solon, OH, USA) and cholic acid was purchased from Santa Cruz Biotechnology (Santa Cruz, CA, USA). Palm oil was purchased from Yee Lee Edible oils Sdn. Bhd. (Perak, Malaysia), and simvastatin and sialic acid (Neu5Ac) were purchased from Pfizer (New York, NY, USA) and Carbosynth Limited (Compton, Berkshire, UK), respectively. ELISA kits (Insulin, adiponectin, CRP, IL-6 and TNF-α) were purchased from Elabscience Biotechnology Co., Ltd (Wuhan, China) while 1,1,3,3-tetramethoxypropane (TMP), thiobarbituric acid, potassium persulfate (K_2_S_2_O_8_), 2,2’-azino-bis[3-ethylbenzothiazoline-6-sulphonic acid] (ABTS) reagent, trolox standard and trichloroacetic acid were purchased from Sigma Aldrich (St. Loius, MO, USA). The RNA extraction and GenomeLab™ GeXP Start Kits were purchased from RBC Bioscience Corp. (Taipei, Taiwan) and Beckman Coulter Inc. (Miami, FL, USA), respectively. Lipid profile, uric acid, alkaline phosphatase (ALP), aspartate transaminase (AST), and alanine transaminase (ALT), urea and creatinine analytical kits were purchased from Randox Laboratories Ltd (Crumlin, County Antrim, UK). RCL2 Solution was purchased from Alphelys (Toulouse, France), while analytical grade ethanol was purchased from Merck (Darmstadt, Germany). All other solvents were of analytical grade and purchased from Merck (Darmstadt, Germany).

### Animal study

Approval for the use of animals for the present study was given by the Animal Care and Use Committee (ACUC) of the Faculty of Medicine and Health Sciences, Universiti Putra Malaysia (Serdang, Selangor, MY), with project approval number: UPM/IACUC/AUP-R011/2014. Animals were handled as stipulated by the guidelines for the use of animals set by the ACUC of the faculty. Ten-week old male Sprague Dawley rats (*n* = 30, body weights between 230–280 g), were housed in individual cages and maintained at room temperature (25 ± 2 °C, 12/12 h light/dark cycle). Rats were acclimatized for 2 weeks with free access to water and normal pellet, and were then divided into 5 groups, (6 rats/group) as shown in Table 1. The normal group received normal rat pellet throughout the 12 weeks of intervention, while the rest of the groups received a high fat diet (HFD) alone, HFD + simvastatin (10 mg/kg/day) [17], HFD + low dose Neu5Ac/SA (50 mg/kg/day) or HFD + high dose Neu5Ac/SA (400 mg/kg/day). Food intake was measured daily while body weights were measured every week. At the end of the intervention, rats were sacrificed after an overnight fast, and their blood and organs were stored for further studies.

**Table 1:** Animal groups, food composition and food intake

Rat groups	Normal pellet (%)	Cholesterol/cholic acid (%)	Palm oil (%)	Starch (%)	Others	Food intake	
						g/kg/day	Kcal/kg/day
Normal	100					64.34 ± 10.96	215.54 ± 33.5^a^
Untreated control	65	5	20	10		48 ± 8.36	215.04 ± 37.45^a^
(HFD)							
HFD + SIM	65	5	20	10	Simvastatin (10 mg/kg/day)	48.14 ± 8.17	215.67 ± 36.60^a^
HFD + SAL	65	5	20	10	50 mg/kg/day sialic acid	48.25 ± 8.32	216.16 ± 37.27^a^
HFD + SAH	65	5	20	10	400 mg/kg/day sialic acid	48.11 ± 8.12	215.53 ± 36.38^a^

Data represent mean ± SD (*n* = 6). Different alphabets in each column indicate significant difference (*p* < 0.05) using Tukey’s multiple comparison test. *HFD*: high fat diet; *HFD + SAL*: HFD+ 50 mg sialic acid/kg/day; *HFD + SAH*: HFD + 400 mg sialic acid/kg/day

### Lipid profile, liver enzymes, uric acid, urea and creatinine

Serum lipid profile, liver enzymes (ALP, AST, and ALT), uric acid, urea and creatinine analyses were performed using Randox analytical kits according to manufacturer’s instructions on a Selectra XL instrument (Vita Scientific, Dieren, The Netherlands).

### Oral glucose tolerance test (OGTT), serum insulin and adiponectin, and homeostatic model of insulin resistance (HOMA-IR)

OGTT was done after an overnight fast using a glucometer (Roche Diagnostics, Indianapolis, IN, USA), while serum insulin and adiponectin levels were measured using ELISA kits according to the manufacturer’s instructions. The absorbance for ELISA analysis was read on a BioTeK Synergy H1 Hybrid Reader (BioTek Instruments Inc., Winooski, VT, USA) at 450 nm and the results were analyzed on www.myassays.com using four parametric test curve (Insulin: R^2^ = 1; adiponectin: R^2^ = 0.99). Furthermore, HOMA-IR was computed from the fasting plasma glucose and insulin levels using the formula, HOMA-IR = (fasting glucose level [mg/dL]/fasting insulin [uU/mL])/2430 [18].

### Serum C-reactive protein (CRP), interleukin 6 (IL-6) and tumor necrosis factor alpha (TNF-α)

Serum CRP, IL-6 and TNF-α were measured using the respective ELISA kits according to the manufacturers’ instructions. Absorbance was read on BioTeK Synergy H1 Hybrid Reader (BioTek Instruments Inc., Winooski, VT, USA) at 450 nm. Results were computed into fold changes by dividing the results of the different groups with those of the normal group.

### Serum total antioxidant status

Total antioxidant status was measured using ABTS radical scavenging activity assay. Briefly, 2.45 mM potassium persulfate (K_2_S_2_O_8_) solution was prepared using 6.62 mg K_2_S_2_O_8_ dissolved in 10 mL of distilled water, while 7 mM ABTS solution was prepared by dissolving 38.4 mg ABTS in 10 mL distilled water. The two solutions were mixed and incubated for 16 h in the dark. The mixture was then diluted using distilled water to obtain a spectrophotometric absorbance of 0.700 ± 0.005 at 735 nm. Furthermore, serum samples (100 μL) or trolox (100 μL) were mixed with 900 μL of the diluted ABTS solution, and their absorbance read at 735 nm. The ABTS radical cation scavenging activity was expressed as the percentage reduction in absorbance.

### Liver Thiobarbituric acid reactive species (TBARS)

Liver tissue TBARS was measured as reported by Chan et al. [19] with minor modifications. Briefly, liver tissue (80 mg) was homogenized in 250 μL of 0.25Hcl, 250 μL of 0.375 % thiobarbituric acid and 250 μL of 15 % tricholoroacetic acid, and the mixture was incubated at 100 °C for 10 min. Subsequently, the mixture was cooled down and centrifuged at 3,000 rpm for 15 min. Finally, 100 μL of each sample and standard (TMP) was loaded into a 96-well plate and the absorbance was read at 532 nm using BioTeK Synergy H1 Hybrid Reader (BioTek Instruments Inc., Winooski, VT, USA). Results were expressed as μM MDA/mg tissue.

### Gene expression study

Primers were designed on the National Center for Biotechnology Information website (http://www.ncbi.nlm.nih.gov/nucleotide/) using the *Rattus norvegicus* gene sequences. All primer sequences were tagged with 18-nucleotide universal forward and 19-nucleotide universal reverse sequences, respectively. Primers (Table 2) were supplied by Integrated DNA Technologies (Singapore), and stored at −20 °C prior to using them for the gene expression study. Hepatic RNA was extracted using the Total RNA extraction kit (RBC Bioscience Corp., Taipei, Taiwan) according to the manufacturer’s instructions, and was used (20 ng/mL) for reverse-transcription and PCR according to the GenomeLab™ GeXP Start Kit protocol (Beckman Coulter, USA), as detailed in Table 2. Then, 1 μL of the PCR products was mixed with sample loading buffer and DNA size standard-400 and analyzed on the GeXP genomelab genetic analysis system (Beckman Coulter, Inc, Miami, FL, USA). The gene expression results were finally normalized using the Fragment Analysis module of the GeXP system software and the eXpress Profiler software.

**Table 2:** Names, accession numbers and primer sequences used in the study

Name	Left sequence	Right sequence
nfkb1	AGGTGACACTATAGAATACACTCCATATTTAATGCAGA	GTACGACTCACTATAGGGAGAAATCCTCTCTGTTTCG
B2m^a^	AGGTGACACTATAGAATAATGCTTGCAGAGTTAAACA	GTACGACTCACTATAGGGATGCATAAAATATTTAAGGTAAGA
Hprt1^a, b^	AGGTGACACTATAGAATATCCTCATGGACTGATTATG	GTACGACTCACTATAGGGACTGGTCATTACAGTAGCTCTT
Rpl13a^a^	AGGTGACACTATAGAATAATGGGATCCCTCCAC	GTACGACTCACTATAGGGAATTTTCTTCTCCACATTCTT
Kan(r)^c^		
Sod1	AGGTGACACTATAGAATAATATGGGGACAATACACAA	GTACGACTCACTATAGGGATCCAACATGCCTCTCT
Sod2	AGGTGACACTATAGAATACAGGTTGCTCTTCAGC	GTACGACTCACTATAGGGAAACTCTCCTTTGGGTTCT
Gpx1	AGGTGACACTATAGAATATTGAGAAGTTCCTGGTAGGT	GTACGACTCACTATAGGGATTTTCTGGAAATCAGGTGT
Gsr	AGGTGACACTATAGAATAAATAAACTGGGGATTCAGAC	GTACGACTCACTATAGGGAAGTAGATTTTCACATTGTCTTTG
CRP	AGGTGACACTATAGAATACTAAACAGGCCTTCGTATT	GTACGACTCACTATAGGGACAAGCCAAAGCTCTACAAT

^a^Housekeeping genes. ^b^Normalization gene. Underlined sequences are left and right universal sequences (tags). ^c^internal control supplied by Beckman Coulter Inc (Miami, FL, USA) as part of the GeXP kit. RT conditions were: 48 °C for 1 min; 37 °C for 5 min; 42 °C for 60 min; 95 °C for 5 min, then hold at 4 °C. PCR conditions were initial denaturation at 95 °C for 10 min, followed by two-step cycles of 94 °C for 30 s and 55 °C for 30 s, ending in a single extension cycle of 68 °C for 1 min

### Data analysis

The data expressed as means ± standard deviation were analyzed using one-way analysis of variance (ANOVA) with Tukey Post-hoc comparisons on SPSS 17.0 software (SPSS Inc., Chicago, IL, USA). Here, *p* < 0.05 was considered statistically significant.

## Results and discussions

### Effects of Neu5Ac on food intake and body weights

**Fig. 1:**
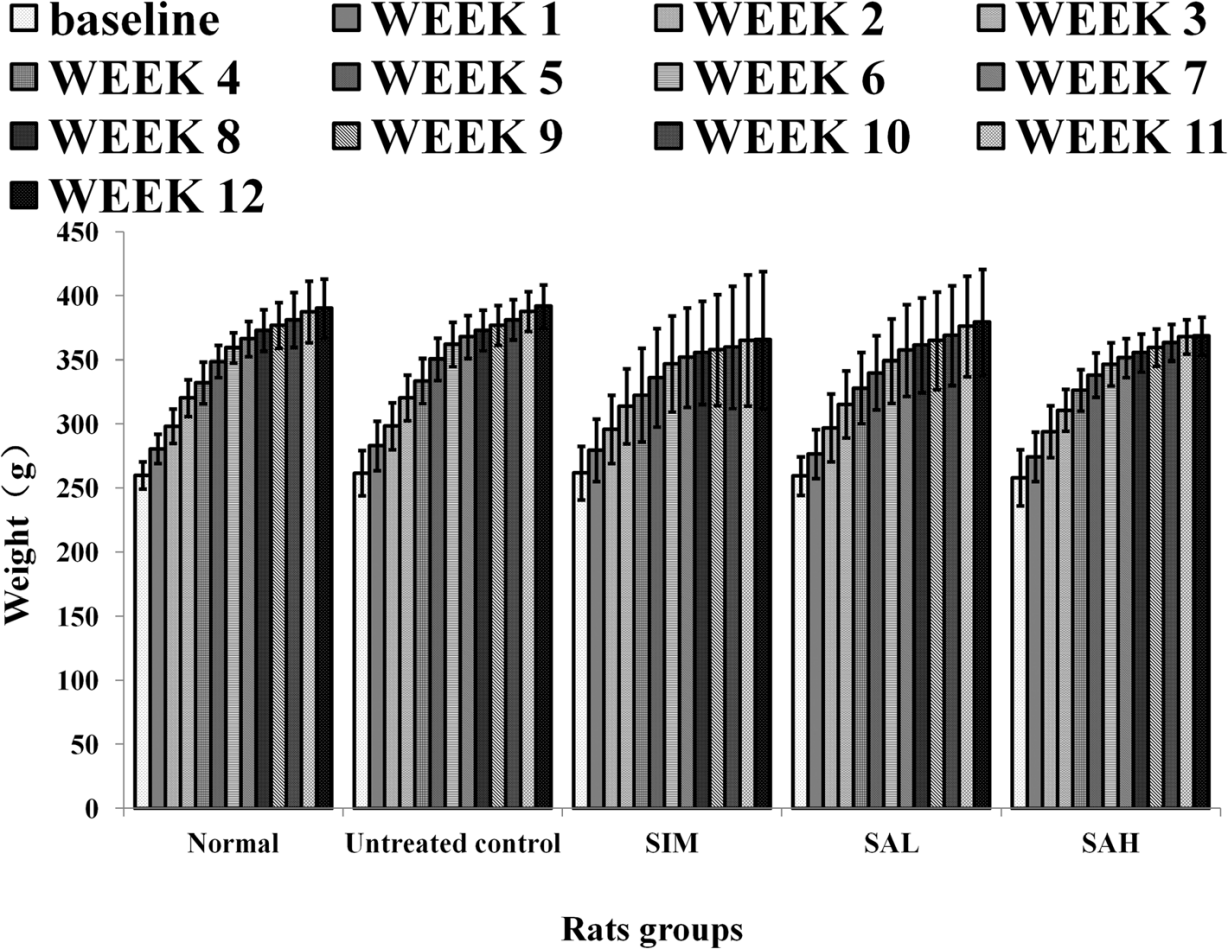
Body weight changes in high fat diet-fed rats over 12 weeks of intervention. Groups are the same as Table 1

Food intake remained similar between the groups throughout the period of intervention (Table 1). And although there were no significant differences observed in the body weights of the different groups (Fig. 1), the untreated control (HFD) group had the highest body weight gain in comparison with the other groups. Moreover, body weight gain is an important factor in the development of oxidative stress, which helps to explain the association between oxidative stress and obesity [20]. The high dose SA (SAH) group showed the lowest weight gain, suggesting that Neu5AC could have some influence on HFD-induced weight gain. Dietary supplementation with Neu5AC could have influenced weight through differential sialylation of glycoprotein structures across the body [11].

### Effects of Neu5Ac on lipid profile, serum insulin and adiponectin, and insulin resistance index

**Fig. 2:**
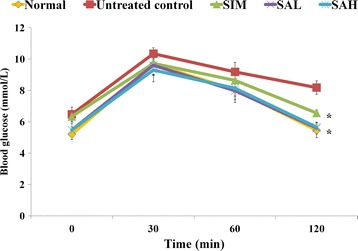
Oral glucose tolerance test in high fat diet-fed rats after 12 weeks of intervention. Groups are the same as Table 1. ^*^ indicates statistically significant difference (*p* < 0.05) in comparison with the untreated (high fat diet) group

Dyslipidemia and insulin resistance are associated with an increased risk of cardiovascular disease, while improvements in these factors are reported to prevent cardiovascular disease [21]. In this study, HFD feeding worsened lipid profiles (Table 3) and insulin resistance (HOMA-IR [Table 3] and OGTT [Fig. 2]) although serum insulin levels remained the same for the groups (Table 3), while supplementation with Neu5Ac attenuated the HFD-induced changes. However, among the lipid profiles, only triglycerides were significantly lower in the Neu5Ac groups in comparison with the HFD group. Worsening of lipid profiles, as seen in the untreated control group, is linked to cardiometabolic diseases, while improved levels are important therapeutic and preventive measures [21]. Additionally, the OGTT results for the Neu5Ac-treated groups showed significantly better insulin sensitivity in comparison with the normal, simvastatin and HFD groups (Fig. 2). Serum adiponectin levels were reduced in the untreated control group (Table 3), in keeping with increased risk of cardiometabolic diseases [22]. Although simvastatin improved lipid profiles, it worsened serum adiponectin levels, which partly explains the basis for the worsening of metabolic indices and increased risk of insulin resistance due to simvastatin [23]. Moreover, adiponectin is an important regulator of metabolism, and has been shown to modulate various pathways with resultant improvements in metabolic outcomes, while its reduction has been linked with worsening cardiometabolic outcomes [22]. Overall, the improved lipid profiles, serum adiponectin and insulin sensitivity in the Neu5Ac groups is indicative of the potential of Neu5Ac supplementation to prevent HFD-induced metabolic perturbations and cardiovascular diseases. As suggested earlier, these changes may have been due to differential sialylation of glycoproteins with consequent implications for different metabolic pathways [24, 25].

**Table 3:** Lipid profile and insulin resistance index

Rat groups	Chol. (mmol/L)	Trig. (mmol/L)	LDL (mmol/L)	HDL (mmol/L)	Insulin (pg/mL)	HOMA-IR	Adiponectin (ng/mL)
Normal	1.55 ± 0.43^a^	0.62 ± 0.15^a^	0.28 ± 0.11^a^	1.18 ± 0.35^a^	495 ± 51.3^a^	1.91 ± 0.23^a^	72.9 ± 0.7^a^
Untreated control	7.47 ± 1.13^b^	1.21 ± 0.38^b^	4.98 ± 1.03^b^	1.05 ± 0.13^a^	513.3 ± 38.8^a^	2.46 ± 0.22^b^	61.8 ± 6.8^b^
(HFD)							
HFD + SIM	4.99 ± 1.11^c^	0.63 ± 0.18^a^	3.6 ± 1.1^b^	1.04 ± 0.17^a^	602.1 ± 145.7^a^	2.83 ± 0.79^a,b^	53.3 ± 0.4^c^
HFD + SAL	5.68 ± 2.18^b,c^	0.54 ± 0.07^a^	4.48 ± 1.81^b^	1.04 ± 0.28^a^	521.25 ± 118.65^a^	2.12 ± 0.56^a,b^	68.1 ± 1.0^b,d^
HFD + SAH	5.05 ± 2.07^b,c^	0.54 ± 0.07^a^	3.67 ± 1.58^b^	1.08 ± 0.27^a^	512.77 ± 90.3^a^	2.08 ± 0.42^a,b^	73.9 ± 5.8^a,d^

Data represent mean ± SD (*n* = 6). The high fat diet-induced hypercholesterolemia attenuated by sialic acid supplementation, although it was only statistically different for the triglycerides (*p* < 0.05). Serum insulin levels were similar among all groups, but the insulin resistance index (HOMA-IR) indicated better insulin sensitivity in the sialic acid groups in comparison with the untreated control group. High fat diet-induced hypoadiponectinemia was also attenuated by sialic acid supplementation. For each parameter in a column, different superscript letters indicate statistical difference between any 2 groups (*p* < 0.05) using Tukey’s multiple comparison test. Groups are the same as Table 1. *HDL*: high-density lipoprotein; *HFD*: high fat diet; *HOMA-IR*: homeostatic model assessment of insulin resistance; *LDL*: low-density lipoprotein; *Chol*: cholesterol; *SAH*: high dose sialic acid; *SAL*: low dose sialic acid; *SIM*: Simvastatin; *Trig*: triacylglycerol

### Effects of Neu5Ac on liver enzymes, urea and creatinine

**Fig. 3:**
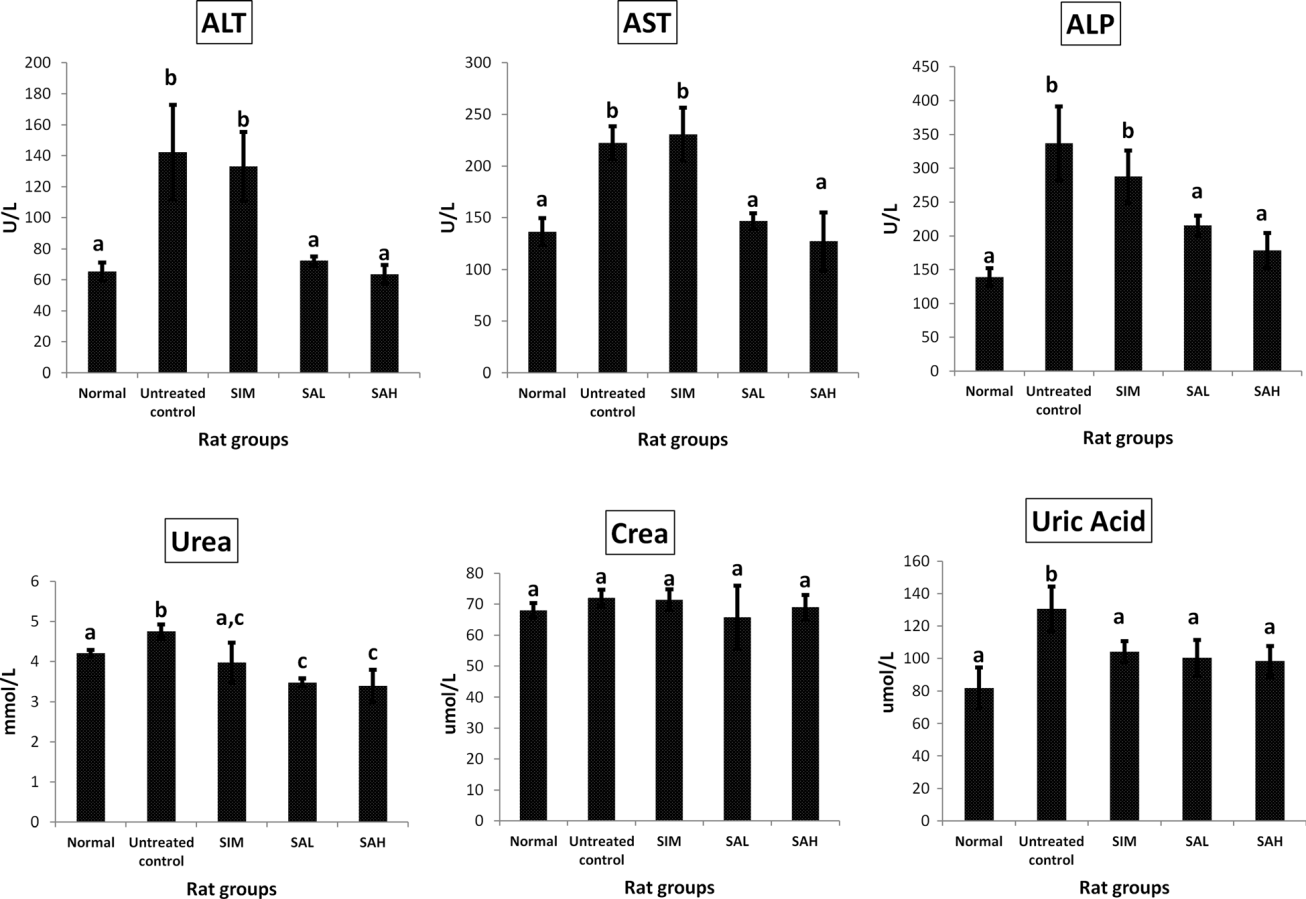
Serum alanine transaminase (ALT), aspartate transaminase (AST), alkaline phosphatase (ALP), urea, creatinine and uric acid in high fat diet-fed rats after 12 weeks of intervention. Groups are the same as Table 1. ^*^ indicates statistically significant difference in comparison with the untreated (high fat diet) group (*p* < 0.05) in each panel

Liver enzymes, kidney function markers (urea ad creatinine) and serum uric acid are shown in Fig. 3. HFD feeding worsened serum AST, ALT, ALP, urea and uric acid levels, while creatinine remained the same. The levels of these markers were significantly improved by supplementation with Neu5Ac and were comparable to the normal group. Interestingly, simvastatin improved lipid profiles but the data showed that it did not improve liver or kidney functions. The roles of the liver and kidneys in maintaining homeostasis are well established. These organs are the targets of cardiometabolic perturbations and their affectation has been shown to further propagate these diseases [26, 27]. The worsened liver and kidney functions in the untreated and simvastatin groups suggested deterioration in their functions, while the improved markers in the Neu5Ac groups indicated that its supplementation could protect or assist in the regeneration of hepatocytes.

### Effects of Neu5Ac on serum markers of inflammation and oxidative stress

**Fig. 4:**
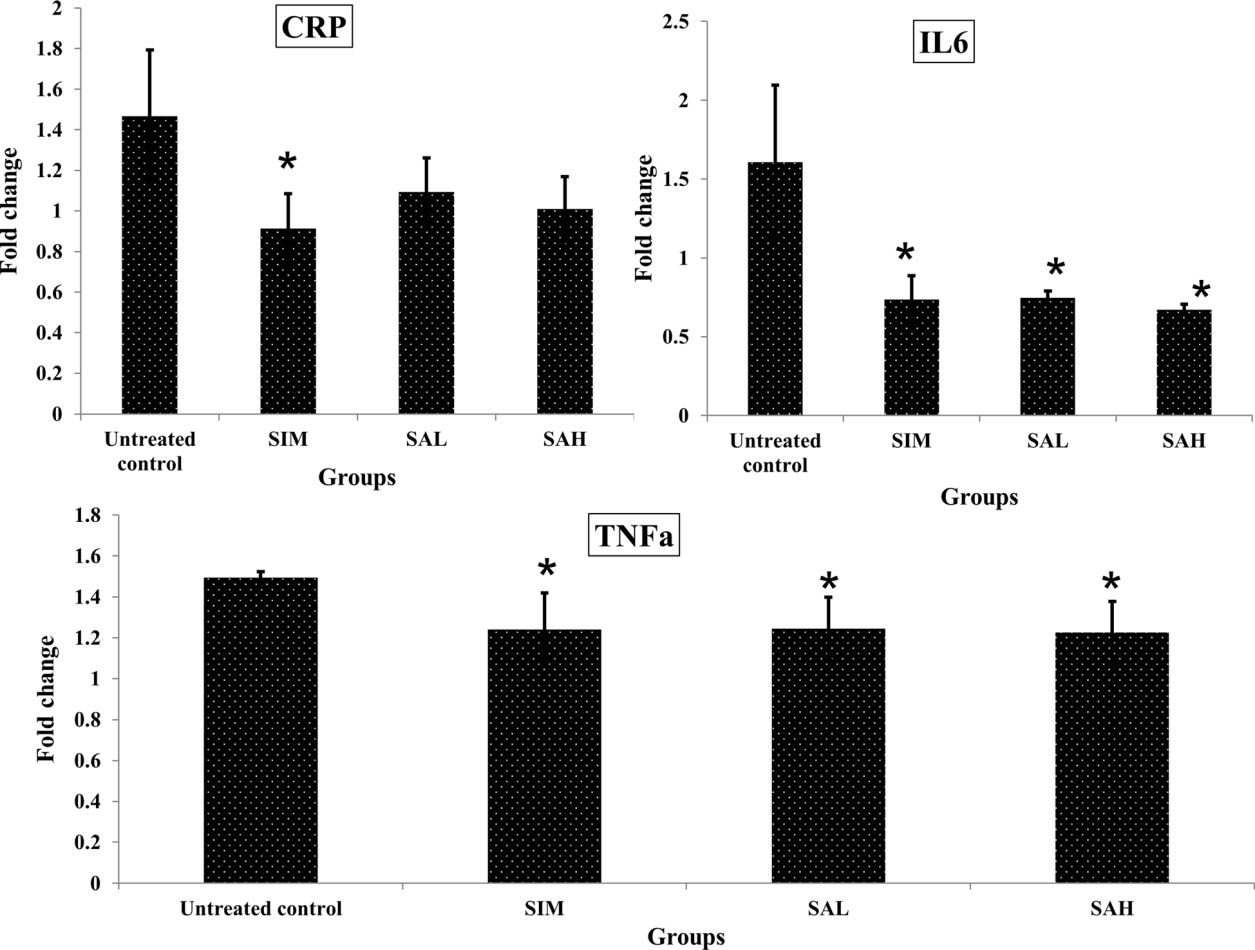
Serum markers of inflammation (CRP:C-reactive protein; IL6:interleukin 6; TNF-α:tumor necrosis factor alpha) in high fat diet-fed rats after 12 weeks of intervention. Groups are the same as Table 1. ^*^ indicates statistically significant difference in comparison with the untreated (high fat diet) group (*p* < 0.05) in each panel

Inflammatory markers such as serum CRP, IL-6 and TNF-α have a significant relationship with cardiovascular disease [28]. In this study, there were significant increases in serum CRP, IL-6 and TNF-α in the HFD group compared with the simvastatin and Neu5Ac groups (Fig. 4). Simvastatin and Neu5AC showed modest reductions in inflammatory markers, although only simvastatin significantly reduced CRP, while both interventions (simvastatin and Neu5AC) reduced IL-6 and TNF-α significantly to similar degrees. Inflammation is reported to promote cardiovascular disease, although there have been suggestions that cardiovascular disease may in fact be a manifestation of inflammation [28], which can be induced by the consumption of a high fat diet [29]. Simvastatin has previously been shown to possess anti-inflammatory effects [30], and the present results showed that dietary Neu5AC may have similar potentials for the attenuation of inflammation.

**Fig. 5:**
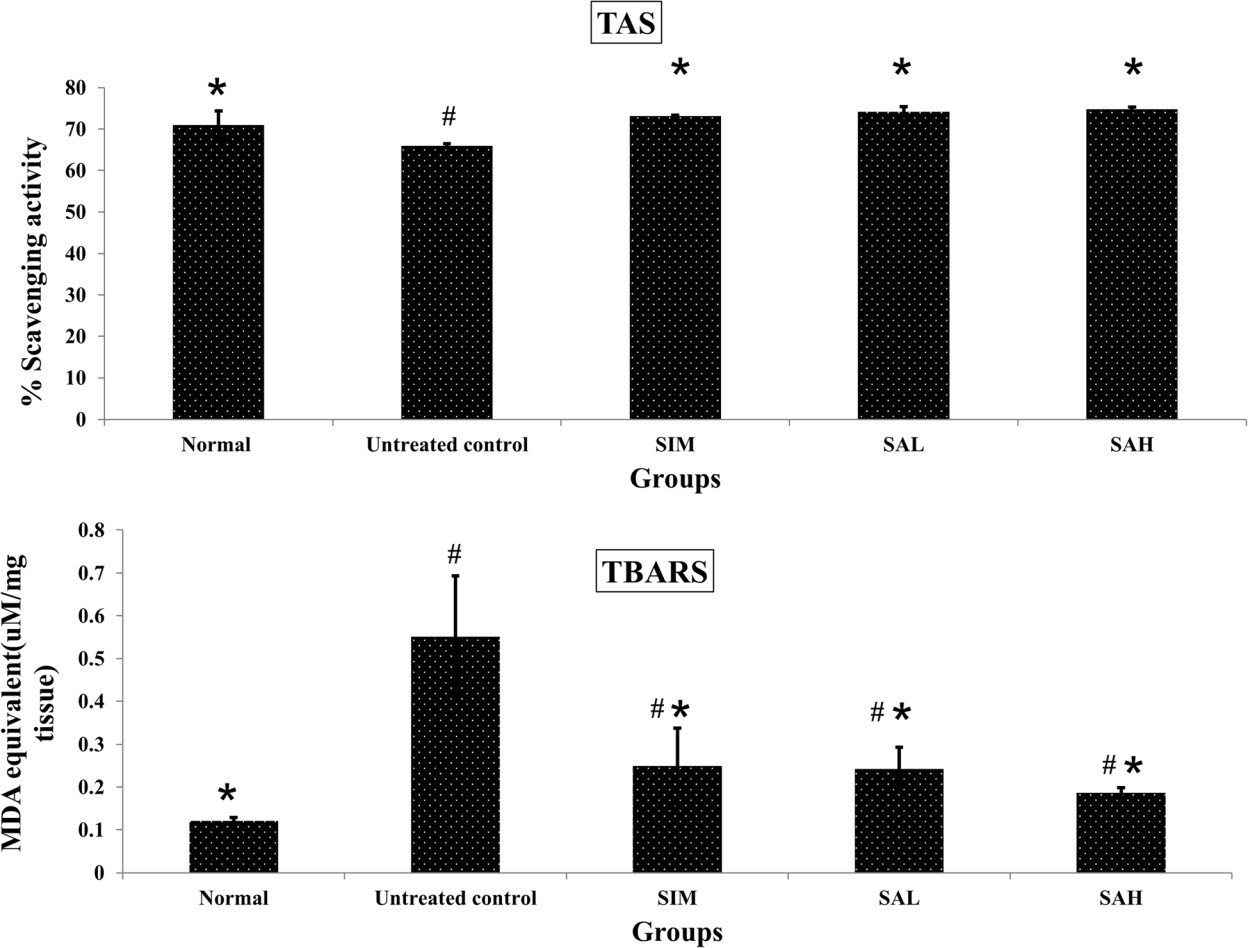
Oxidative stress markers (serum TAS:total antioxidant status; liver TBARS:thiobarbituric acid reactive species) in high fat diet-fed rats after 12 weeks of intervention. Groups are the same as Table 1. ^*^indicates statistical difference in comparison with the untreated (high fat diet) group (*p* < 0.05) in each panel. ^#^indicates statistically significant difference in comparison with the normal group (*p* < 0.05) in each panel

Furthermore, markers of oxidative stress were worsened in the HFD group (Fig. 5), as suggested by the increased TBARS and decreased TAS in comparison with the normal group. Simvastatin and Neu5AC, on the other hand, improved oxidative stress significantly by increasing TAS and reducing TBARS. The results suggested that HFD may promote oxidative stress and inflammation in addition to weight gain, dyslipidemia and insulin resistance, while simvastatin and Neu5AC will prevent such HFD-induced changes. The effects of simvastatin have been reported previously [30] but this is the first demonstration of such effects for Neu5AC supplementation.

### Effects of Neu5Ac on mRNA levels of hepatic antioxidant and inflammation genes

**Fig. 6:**
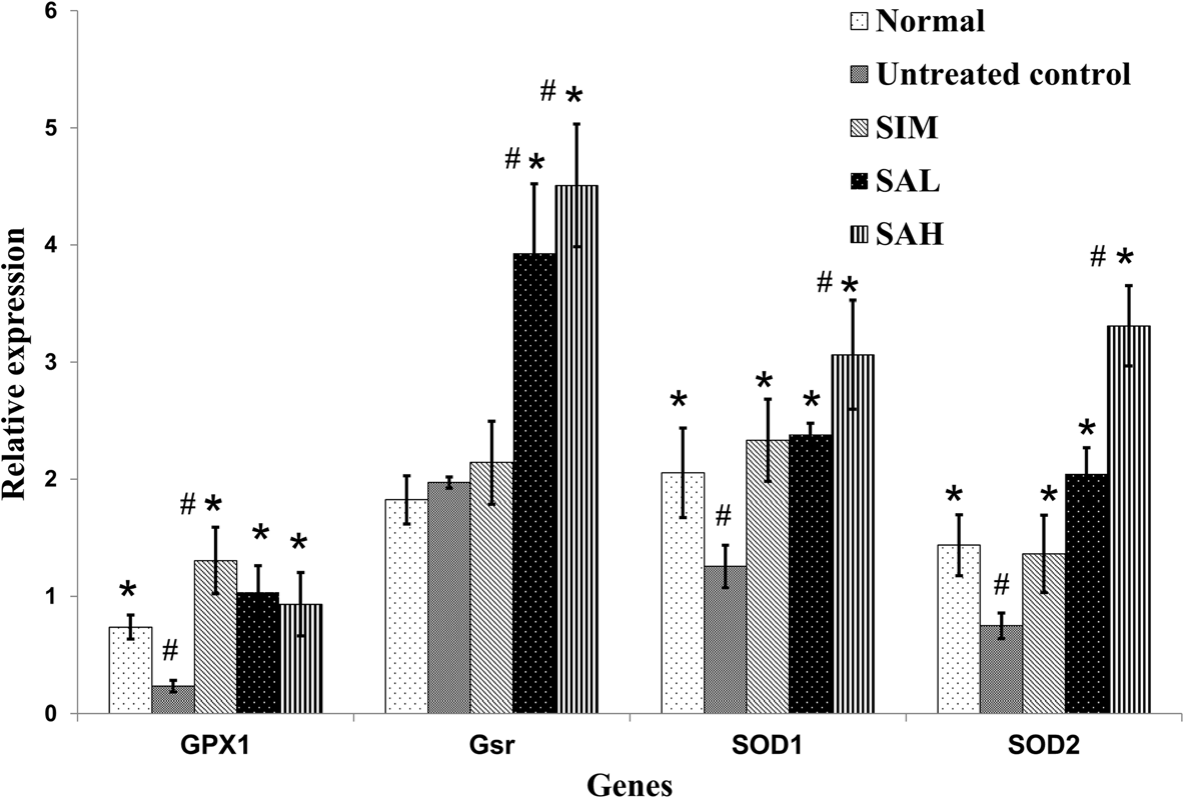
Hepatic mRNA levels of glutathione peroxidase (Gpx), glutathione reductase (Gsr), superoxide dismutase (SOD) 1 and 2 in high fat diet-fed rats after 12 weeks of intervention. Groups are the same as Table 1. ^*^indicates statistically significant difference in comparison with the untreated (high fat diet) group (*p* < 0.05) for each gene. ^#^indicates statistically significant difference in comparison with the normal group (*p* < 0.05) for each gene

**Fig. 7:**
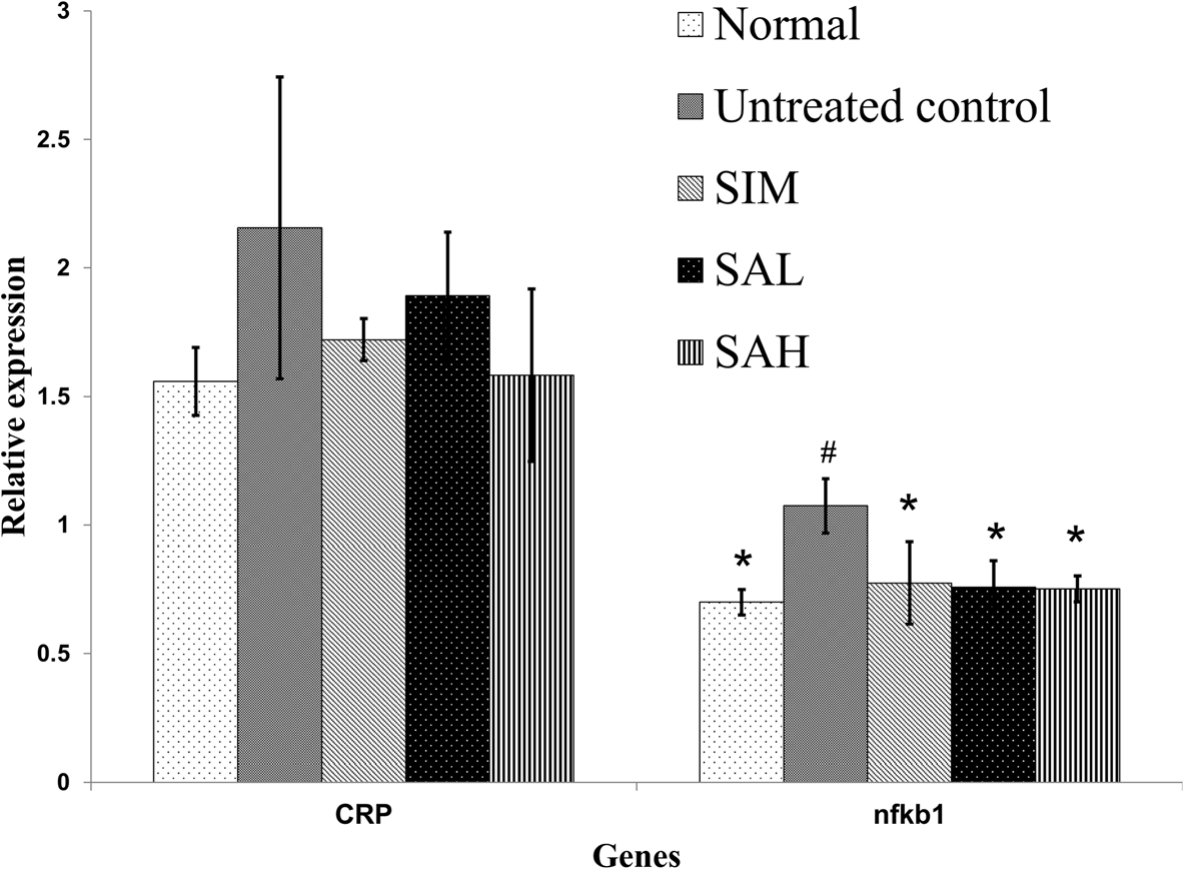
Hepatic mRNA levels of C-reactive protein (CRP) and nuclear factor kappa beta (nfkb) in high fat diet-fed rats after 12 weeks of intervention. Groups are the same as Table 1. ^*^indicates statistically significant difference in comparison with the untreated (high fat diet) group (*p* < 0.05). ^#^indicates statistically significant difference in comparison with the normal group (*p* < 0.05) for each gene

Based on the observations that Neu5AC possessed antioxidative and anti-inflammatory effects, we studied the potential mechanistic bases for such effects by measuring the mRNA levels of related hepatic genes (Figs. 6 and 7). The results indicated that HFD feeding decreased mRNA levels of hepatic antioxidant genes (glutathione peroxidase [Gpx], and superoxide dismutase [SOD]) (Fig. 6) and increased those of inflammatory genes (CRP and nuclear factor kappa beta [nfkb]) (Fig. 7). Although simvastatin and Neu5AC improved mRNA levels of hepatic antioxidant genes, Neu5AC had more profound effects, especially at higher doses (glutathione reductase [Gsr] and SOD). Additionally, simvastatin and Neu5AC produced lower mRNA levels of inflammatory genes, although the CRP gene expression was not significantly different when compared with the HFD group. However, the mRNA levels of nfkb1 gene in the simvastatin and Neu5AC groups were significantly different when compared with the HFD group.

The effects of simvastatin observed in the present study are in keeping with its reported antioxidant and anti-inflammatory effects [30]. However, the effects of simvastatin on liver and kidney functions and insulin sensitivity indicated that it was not suitable for long-term use [23]. Neu5AC supplementation, on the other hand, has not been previously reported to reduce inflammation and oxidative stress induced by HFD feeding. The present results, therefore, demonstrated for the first time that Neu5Ac supplementation could prevent HFD-induced metabolic perturbations, partly through transcriptional regulation of related hepatic genes. Disparity between gene expression results and serum biochemical markers like those of CRP could indicate that Neu5AC and simvastatin modulate their effects at transcriptional and non-transcriptional levels, or that other post-transcriptional modifications are involved in their overall effects.

## Conclusions

We demonstrated for the first time that dietary Neu5Ac supplementation attenuated HFD-induced weight gain, dyslipidemia, insulin resistance, inflammation and oxidative stress, partly through the transcriptional regulation of related hepatic genes in rats. In view of the side effect profile of most pharmaceuticals currently in use, the present results suggest that Neu5AC supplementation may be used as a safer alternative for the management of diseases in which obesity, inflammation and oxidative stress are central problems, including cardiometabolic diseases. However, further studies are indicated to establish the long term effects of Neu5Ac supplementation and its translational implications.

## Abbreviations

HFD: High fat diet
CRP: C-reactive protein
IL6: Interleukin-6
Tnf-α: Tumor necrosis factor-alpha
ABTS: 2,2’-azino-bis[3-ethylbenzothiazoline-6-sulphonic acid]
TBARS: Total antioxidant status
Gpx: Glutathione peroxidase
Gsr: Glutathione reductase
SOD1: Superoxide dismutase 1
SOD2: Superoxide dismutase 2
Ccl2: Pyruvate kinase
Neu5Gc: N-glycolylneuraminic acid
Neu5Ac: N-acetylneuraminic acid
